# Occupational exposure to wood dust and risk of lung cancer in two population-based case–control studies in Montreal, Canada

**DOI:** 10.1186/1476-069X-14-1

**Published:** 2015-01-07

**Authors:** Eric Vallières, Javier Pintos, Marie-Elise Parent, Jack Siemiatycki

**Affiliations:** INRS – Armand-Frappier Institute, Université du Québec, Laval, QC Canada; CHUM Research Center, Université de Montréal, Montréal, QC Canada; Department of Social and Preventive Medicine, University of Montreal, Montreal, QC Canada

**Keywords:** Wood dust, Lung cancer, Epidemiology, Case–control studies, Occupational exposure, Tobacco

## Abstract

**Background:**

Wood dust is one of the oldest and one of the most common occupational exposures in the world. The present analyses examine the effect of lifetime exposure to wood dust in diverse occupational settings on lung cancer risk.

**Methods:**

We conducted two population-based case–control studies in Montreal: Study I (1979–1986) included 857 cases and two sets of controls (533 population and 1349 cancer controls), and Study II (1996–2001) comprised 736 cases and 894 population controls. Detailed job histories were obtained by interview and each job was evaluated by expert chemist–hygienists to estimate the likelihood and level of exposure to many substances, one of which was wood dust. Odds ratios (ORs) were computed in relation to different indices of exposure to wood dust, adjusting for several covariates including smoking. Three datasets were analysed: Study I with population controls, Study I with cancer controls, and Study II.

**Results:**

The most frequently exposed occupations in our study population were in construction, timber and furniture making industries. We found increased risks of lung cancer for substantial cumulative exposure to wood dust in Study I with cancer controls, (OR = 1.4: 95% confidence interval 1.0;-2.0) and in Study II (OR = 1.7: 95% confidence interval 1.1-2.7). There were no excess risks of lung cancer in any of the three datasets among workers whose cumulative exposure was not substantial. These tendencies held equally within strata of low smokers and heavy smokers.

**Conclusion:**

There was evidence of increased risk of lung cancer among workers with substantial cumulative exposure to wood dust.

**Electronic supplementary material:**

The online version of this article (doi:10.1186/1476-069X-14-1) contains supplementary material, which is available to authorized users.

## Background

Lung cancer is one the most common and lethal malignancies worldwide, resulting in over one million deaths each year [1]. Although tobacco smoking is by far the main determinant of lung cancer, accounting for approximately 90% of the cases among men, environmental and occupational exposures also contribute greatly, with the estimated attributable fraction varying from 5% to 15% [2, 3].

Wood dust is one of the most common occupational exposures, with millions of workers exposed worldwide [4]. This complex substance is mainly composed of cellulose (40%-50%), polyoses and other substances, but its exact formula depends on the species of tree being processed [5]. Trees are characterized as gymnosperms (softwoods), and angiosperms (hardwoods), with the latter being generally denser and producing finer and more abundant dusts. The amount and size of particles also differ according to the operations performed on wood namely, shattering wood cells during sanding operations produces finer particle size than does chipping in sawing and milling industries [6].

In 1995, the International Agency for Research on Cancer (IARC) classified wood dust as carcinogenic to humans (group 1), mainly based on findings for cancers of the nasal cavities and paranasal sinuses. For lung cancer, the evidence for an association was inconsistent across studies. Since then, several additional epidemiological studies have been published, and IARC conducted a new evaluation in 2009 [5]. While that evaluation reiterated a Group 1 classification for wood dust in relation to cancers of the nasopharynx and nasal cavity, the evidence for lung cancer was judged as weaker, based on inconsistent findings.

Some studies found an increased risk of lung cancer in specific occupations and industries, such as sawmill workers [6, 7] and carpenters [8, 9], while others found no association among pulp and paper mill workers [10] or furniture workers [11]. Exposure to wood dust, when assessed across a wide range of occupations, was found not to be associated [12, 13] or to confer a slight excess risk of lung cancer [14–17]. However, some of these studies could not account for potential confounders such as tobacco smoking and exposure to asbestos.

In the early 1980s we conducted a population-based case–control study in Montreal, Canada, to explore possible associations between hundreds of occupational substances and multiple cancer sites, including lung cancer (Study I). In the late 1990s, we carried out a similar study in the same area, this time focusing on lung cancer (Study II). The present set of analyses, conducted in both studies, examines the risk of lung cancer associated with occupational exposure to wood dust, while controlling for major confounders, including smoking and other occupational exposures. An initial analysis of Study I concerning the association between wood dust and several sites of cancer, including lung, was previously published [18]. It is analyzed here in more detail, as is the data from Study II, to be able to contrast results from the two studies, and more importantly to provide more valid and comprehensive results than those previously published. Namely, the present analysis of Study I improves on the previous analysis in several regards. First, there have been re-evaluations of exposure assessments to some subject files. Second, the previous publication only reported results using cancer controls; this one reports results using both the cancer and population controls. Third we have improved the statistical modeling of confounders, most notably smoking history.

## Methods

Study I was conducted from 1979 to 1986 and included men aged 35–70 years diagnosed with cancer at any of 19 sites [19, 20]. Study II was conducted between 1996 and 2001 and included both men and women aged 35–75 diagnosed with a lung malignancy. Both studies included patients with incident histologically confirmed cancers identified across all major Montreal area hospitals, living in the Montreal area, and restricted to Canadian citizens. Both studies also included a series of population controls randomly selected from electoral lists. Controls were frequency matched by age, sex (only applicable to Study II) and area of residence (electoral district of about 40,000 individuals) to all cancer cases for Study I and to lung cancer cases for Study II. Additional details about subject ascertainment and data collection have been presented previously [19, 21]. Results are presented here for men only, because the prevalence of occupational exposure to wood dust among women was very low in our study population (2%).

Study I included lung cancer cases, other cancer cases and population controls. In computing relative risk estimates for lung cancer we were thus abIe to use as referents not only the population controls, but also the patients with other types of cancer (cancer controls). There are different pros and cons associated with population controls and cancer controls [20, 22]. Although a population-based control group is often considered to be more representative of the base population, cancer controls are less susceptible to non-participation bias and information bias [21]. We cannot affirm that one control group is necessarily more valid than the other in representing the exposure experience of the study base. In study I, 1082 lung cancer cases and 740 eligible population controls were identified and attempts were made to interview them. Of these, 857 (79%) cases and 533 (72%) population controls completed the interview. From the pool of other cancer patients, we selected a set of controls comprising a total of 1349 patients with cancer at a site non-contiguous to the lung, who had been ascertained in the same year and hospitals as the lung cancer cases, and selected so that none of the 19 individual cancer sites represented more than 20% of the overall pool of cancer controls. The main cancer sites in the cancer control series were bladder (17%), colon (15%), prostate (15%), stomach (9%), lymphomas (7%), kidney (6%), and rectum (5%). In study II, 860 eligible male cases and 1294 eligible male controls were identified, and 736 (86%) and 894 (69%) of these, respectively, agreed to participate and satisfactorily completed the interview. Ethical approval was obtained for both studies from the Institut National de la Recherche Scientifique, McGill University and each participating hospital. All participating subjects provided informed consent.

### Data collection

In study I and study II, over 82% and 76% of individuals, respectively, responded for themselves, and surrogate respondents (proxies) provided information for the other participants. Interviews included a structured section that requested information on socio-demographic and lifestyle characteristics, including ethnicity, family income and smoking history, and a semi-structured section that elicited a detailed description of each job held by the subjects in his working lifetime. Occupations were coded according to the 1971 Canadian Classification and Dictionary of Occupations [23]. For each job held, a trained interviewer asked the subject about the company, its product, the nature of the work site, the subject’s main and subsidiary tasks, and any additional information (e.g., equipment maintenance, use of protective equipment, activities of co-workers) that could provide clues about work exposures and their intensity. Supplementary questionnaires were used to assist interviewers with detailed technical probing for some occupations, including among others: carpenters, cabinet makers, drivers, insulation workers and plumbers [24]. A team of chemists and industrial hygienists examined each completed questionnaire and translated each job into a list of potential exposures using a checklist of 294 agents. Wood dust was on the checklist. Unfortunately it was impossible to ascertain whether the exposures were to hardwood or softwood dust, so all types of wood dust are combined in this analysis.

Combining the two studies, more than 28000 jobs were evaluated. The final exposure codes attributed to a participant were based on consensus among the coders. Chemical coders were blind with regards to the subject’s disease status. For each substance considered present in each job, the coders noted three dimensions of information, each on a three-point scale: their degree of confidence that the exposure had actually occurred (possible, probable, definite), the frequency of exposure in a normal work week (<5%, 5%-30%, >30% of the time) and the relative level of concentration of the agent (low, medium, high). Unfortunately, it proved impossible to reliably estimate absolute concentration values corresponding to the relative levels coded.

Non-exposure was interpreted as exposure up to the level that can be found in the general environment. For wood dust, there are no specific environmental measurements to establish a background level. Among those considered exposed, benchmark occupational circumstances were established to correspond to low, medium and high concentrations, and each job was coded with respect to these benchmarks. The ‘low’ concentration benchmark comprised construction carpenters and household furniture makers; ‘medium’ concentration benchmark comprised sawmills, lumber yard and laminating shop workers; ‘high’ concentration benchmark comprised hand and belt sanding operations and finishing departments of plywood production. These benchmarks were indicative and the experts were free to score a given job, the industry, the era and the particular characteristics of the workplace and work habits. Although a subject’s job title was certainly a factor in attributing exposure, the details of the subject’s activities were taken into account in assessing the exposure, as well as the industry and the era. More extensive descriptions of the exposure assessment method can be found elsewhere [20, 25, 26].

### Data analysis

Unconditional logistic regression [27] was used to estimate odds ratios (ORs) and their 95% confidence intervals (CIs) for the association between each occupational factor and lung cancer, adjusting for the following *a priori* potential confounders: age, median income in the census tract of residence and individual schooling level as markers of socioeconomic status, ethnic-cultural background (French, Anglo, other), respondent status (self, proxy), ever occupational exposure to asbestos, diesel engine exhaust, formaldehyde, cadmium, chromium IV compounds, nickel compounds, silica dust, and tobacco smoking. After comparison of several parameterizations of the smoking variables in our data sets, we selected the comprehensive smoking index (CSI), which proved to most accurately fit the data and integrates duration, intensity and time since quitting smoking [28]. This index best captures the confounding nature of smoking history since it takes into account the timing of smoking exposure, and not just the duration and intensity.

There is an ongoing debate as to whether it is appropriate to adjust for markers of socioeconomic status (SES) in occupational studies [29–31], with some arguing that SES is a confounder to be adjusted for and others that it is a collider to be omitted from statistical models. It may also be debated whether the inclusion in the models of other occupational carcinogens may constitute a form of over-adjustment. To examine whether inclusion of SES or other occupational carcinogens has the potential to bias the association between wood dust and lung cancer, we conducted a sensitivity analysis in which we compared results on wood dust exposure from four models: i) without adjustment for SES nor for other occupational carcinogens, ii) adjustment for SES but not other occupational carcinogens, iii) adjustment for other occupational carcinogens but not SES, and iv) adjustment for both SES and other occupational carcinogens. The other core covariates remained in all models.

Occupational exposure indices were based on four dimensions of information that were available whenever the experts assigned an exposure to a subject: probability that the exposure took place, concentration, frequency, and years of beginning and ending exposure. Using these dimensions, an *a priori* cumulative exposure index was calculated with the following categories: ‘no exposure’ consisted of never exposed subjects and those for whom the degree of confidence that the exposure actually occurred was coded as just ‘possible’ by the hygienists; the remaining subjects, whose exposure to wood dust was rated as probable or definite, were considered as ‘exposed’ for these analyses. We further subdivided those ‘exposed’ into two exposure groups: ‘substantial exposure’ was assigned to subjects who had been exposed to medium or high concentrations, during more than 5% of their work week, and for 5 years or more, whereas ‘non-substantial exposure’ was assigned to the remaining exposed subjects. Exposures having occurred less than five years previous to the index date were discounted on latency grounds. Other cumulative exposure indices were calculated using different combinations of weights to the exposure dimensions frequency, concentration, duration and latency. None of these indices showed better goodness-of-fit than the simple categories described above so they are not presented here.

Besides treating smoking as an *a priori* confounder, we explored potential effect modification by smoking. Since the number of never smokers among cases was very low, the non-smokers category was supplemented with lifetime low intensity smokers. Operationally, we defined lifetime low intensity smokers as individuals having a CSI value below the 25^th^ percentile on this scale. Because of the way it is constructed [28], the CSI index does not translate easily onto the duration or daily amount of pack-year scale. We can illustrate the amount of smoking in these categories by showing two smoking profiles that would fall on the 25^th^ percentile of the CSI scale, namely: a current smoker who smoked three cigarettes per day during 40 years (with lifetime cumulative exposure of 6 pack-years), or a former smoker who smoked six cigarettes a day for 30 years and quit 10 years ago (with cumulative exposure of 9.8 pack years). Smokers with CSI values above the 25^th^ percentile were considered medium/heavy smokers. To evaluate the statistical significance of the difference in ORs between the two strata of smokers, we carried out an analysis based on all subjects including the two variables, smoking status (binary) and exposure to wood dust (binary), by testing their cross-product term. The continuous CSI variables were maintained as a covariate in the models to avoid any residual confounding within the smoking status strata.

The associations between wood dust and the most prevalent histologic types of lung cancer, namely squamous cell, adenocarcinoma, small cell and large cell, were also evaluated.

## Results

Table 1 shows the distribution of cases and controls according to different socio-demographic characteristics. In both studies, compared to controls, cases were more likely to have French ancestry, had fewer years of education, lived in census tract regions with lower median family income and were more likely to have had a proxy responding for them. As expected, the proportion of current smokers and the intensity of smoking were higher among cases than among controls. It is noteworthy that there is a marked decrease in the proportion of current smokers between study I and II among the controls, reflecting the smoking habits trend of the last decades in North America [32].

**Table 1:** **Selected socio-demographic characteristics of male subjects in the two case–control studies, Montreal, Canada**

		Study I (1979–1986)			Study II (1996–2001)	
Variables	Categories	Population controls	Cancer controls	Cancer cases	Population controls	Cancer cases
		N = 533	N = 1349	N = 857	N = 894	N = 736
Age group (%)	≤55 years	28.0	32.5	27.4	11.9	13.6
	56-65 years	45.2	43.7	50.8	28.6	32.9
	66-75 years	26.8	23.7	21.8	59.5	53.5
Ethnolinguistic group (%)	French	64.2	58.0	69.1	64.4	77.4
	English	14.1	16.1	13.5	6.4	4.6
	Other	21.8	25.9	17.4	29.2	17.9
Schooling (%)	<7 years	20.3	22.3	30.3	24.7	28.0
	7-12 years	56.1	55.2	57.1	48.1	56.2
	≥13 years	23.6	22.5	12.6	27.2	15.8
Median family income*		100	93	84	100	94
Smoking (%)	Never	19.7	17.3	1.5	17.7	2.4
	Current	46.9	58.0	79.9	29.2	67.5
Quit smoking (%)	2-5 years ago	8.8	6.7	7.6	2.8	4.3
	5-10 years ago	7.9	6.2	6.0	6.6	5.8
	>10 years ago	16.7	11.8	5.0	43.7	19.8
Mean pack-years**		49,9	52.3	74.3	50.3	78.5
Respondent (%)	Self	87.4	80.8	70.6	90.3	60.2
	Proxy	12.6	19.2	29.4	9.7	39.8

*Indicator of inter-subject mean of the median family income for census tract of residence, using the study-specific mean value among population controls as the reference value for each study (x 100). Based on the 1981 census for Study I and the 1991 census for Study II.

**Among ever smokers, based on 20 cigarettes per packet.

Wood dust, among all agents assessed, was one of the most prevalent exposures for males in both our studies. In both studies combined, out of a total of 18,304 jobs classified and evaluated for the present analysis, 1,906 (10.4%) were considered as exposed to wood dust. During the period of greatest relevance of this study (1945–1996), the industrial profile of the Montreal area was quite diverse, and changed substantially during these years. Table 2 presents the occupations where most of the exposure to wood dust occurred in our two study samples. To better illustrate the shift in importance of some occupations across our study periods, we differentiated the exposure that happened before and after 1960. The top four categories of occupations on the list, with varying importance depending on the study and period, are carpenters, timber cutting occupations, cabinet and wood furniture makers, and occupations in laboring and other construction trades. Within each study sample, between the two periods there was an increase in construction-related jobs and a decrease in timber cutting and related occupations.

**Table 2:** **Distribution of occupations held by male subjects exposed to wood dust in two distinct periods**

		Study I			Study II	
	All jobs	Jobs before 1960	Jobs after 1960	All jobs	Jobs before 1960	Jobs after 1960
	n = 1017*	n = 766	n = 539	n = 889	n = 494	n = 569
Occupation title	%**	%	%		%	%
Carpenters and related occupations	16,7	15.9	21.5	11.6	9.3	14.1
Occupations in labouring and other elemental work, and other construction trades	13.3	11.7	16.1	19.7	17.5	23.4
Timber cutting and related occupations	10,7	13.8	4.5	9.9	16.4	3.3
Cabinet and wood furniture makers	7.1	6.4	6.9	4.7	4.3	4.7
Painters, paperhangers and related occupations	3,8	4.0	4.3	1,5	1.0	1.9
General workers, farm	3,2	4.3	0.6	1,3	2.4	0.2
Construction electricians and repair workers	2,5	2.9	2.6	4,2	3.8	5.1
Truck drivers	2,1	2.2	1.1	2,9	2.8	3.0
Brick and stone masons and tile setters	1,7	2.2	1.1	1,2	1.6	1.2
Janitors, charworkers and cleaners	1,5	0.5	2.6	3,3	1.4	4.2
Wood processing, except paper pulp	1.3	1.4	0.9	2,2	3.0	1.2
Pipefitting, plumbing and related occupations	0.9	1.0	0.7	3,1	4.3	3.0
All other jobs with wood dust exposure	35.2	33.7	37.1	34,4	32.2	34.7

*Numbers of jobs with exposure to wood dust. Each subject may have been exposed in more than one job. Jobs that overlapped 1960 were included in both time periods; thus the sum of numbers under the two time periods exceeds the total number of jobs.

**Percentage of subjects with wood dust exposure who were in each listed occupation.

Table 3 shows the adjusted ORs between lung cancer and occupational exposure to wood dust for study II and study I, using both control groups. Overall risk estimates were slightly higher in study II than in study I. In study I, when using population controls, the non-statistically-significant ORs were below 1.0. Results of study I with cancer controls and for study II showed results close to null, except for increased risks at the substantial exposure levels (for study I: OR = 1.4, 95% CI = 1.0-2.0; for study II: OR = 1.7, 95% CI = 1.1-2.7).

**Table 3:** **Odds ratio for lung cancer associated with occupational exposure wood dust in two case–control studies**

	Controls	Cases	OR _1_ *	OR _2_ **	95% CI (OR _2_ )	
**Study I**	*Population Controls*					
No exposure	389	630	1.0	1.0	(ref)	
Any level of exposure	144	227	0.8	0.7	0.5	1.0
Any level ≤ 20 years	88	141	0.8	0.7	0.5	1.0
Any level > 20 years	56	86	0.7	0.7	0.4	1.1
Non-substantial level	74	113	0.8	0.7	0.5	1.0
Substantial level	70	114	0.7	0.7	0.5	1.0
**Study I**	*Cancer Controls*					
No exposure	1072	630	1.0	1.0	(ref)	
Any level of exposure	277	227	1.2	1.1	0.9	1.5
Any level ≤ 20 years	179	141	1.2	1.1	0.8	1.5
Any level > 20 years	98	86	1.2	1.2	0.8	1.7
Non-substantial level	161	113	1.0	1.0	0.7	1.3
Substantial level	116	114	1.5	1.4	1.0	2.0
**Study II**	*Population Controls*					
No exposure	640	501	1.0	1.0	(ref)	
Any level of exposure	254	235	1.2	1.1	0.9	1.5
Any level ≤ 20 years	165	139	1.1	1.0	0.8	1.4
Any level > 20 years	89	96	1.4	1.3	0.9	1.9
Non-substantial level	201	167	1.1	1.0	0.7	1.3
Substantial level	53	68	1.9	1.7	1.1	2.7

*adjusted for age, ethnolinguistic group, years of education, median family income, respondent status and cigarette index.

**adjusted for the same covariates as above, as well as IARC Group 1 occupational carcinogens (asbestos, diesel exhaust, formaldehyde, cadmium, chromium VI, nickel and silica).

Additional file 1: Table S1 shows results of a sensitivity analysis in which we excluded and included two covariates, SES and other occupational carcinogens. The table shows that there was not really a great impact of inclusion or exclusion of these covariates on the OR between wood dust and lung cancer. The only discrepancy among analogous estimates was in Study II, for Substantial exposure; the estimate when including other occupational carcinogens was a bit lower than when not including them in the model. Inclusion or exclusion of SES barely affected the OR estimates.

Table 4 shows OR estimates for exposure to wood dust, stratified by smoking status. Among never/low smokers, most of the results were close to null and did not reach statistical significance, with a wide confidence interval due to small number of never-smokers in both studies. Among medium and heavy smokers, we found an increased risk associated with substantial exposure to wood dust in study II (OR = 1.6, 95% CI = 1.0-1.5). When combining the smoking strata, the interaction terms between smoking and wood dust exposure were not significant. Overall, there was no clear evidence of effect-modification between smoking and wood dust exposure.

**Table 4:** **Odds ratio for lung cancer associated with occupational exposure to wood dust, stratified by smoking status, and test for interaction**

	Never-low smokers					Medium-heavy smokers					p-value
	Controls	Cases	OR*	95% CI		Controls	Cases	OR*	95% CI		(interaction)
**Study I**	*Population Controls*					*Population Controls*					
No exposure	162	56	1.0	(ref)		227	574	1.0	(ref)		
Any level of exposure	48	19	0.7	0.3	1.4	96	208	0.7	0.5	1.0	0.714
Non-substantial level	31	15	0.4	0.1	1.2	44	106	0.8	0.5	1.2	0.310
Substantial level	17	4	1.0	0.4	2.8	52	102	0.6	0.4	1.0	0.110
**Study I**	*Cancer controls*					*Cancer controls*					
No exposure	411	56	1.0	(ref)		661	574	1.0	(ref)		
Any level of exposure	83	19	0.9	0.4	1.7	194	208	1.2	0.9	1.5	0.508
Non-substantial level	50	7	0.5	0.2	1.3	111	106	1.0	0.8	1.4	0.790
Substantial level	33	12	1.6	0.7	3.9	83	102	1.3	0.9	1.9	0.235
**Study II**	*Population Controls*					*Population Controls*					
No exposure	269	40	1.0	(ref)		371	461	1.0	(ref)		
Any level of exposure	87	10	0.8	0.3	1.8	167	225	1.2	0.9	1.6	0.196
Non-substantial level	70	5	0.5	0.2	1.3	131	162	1.1	0.8	1.5	0.078
Substantial level	17	5	2.4	0.7	7.9	36	63	1.6	1.0	1.5	0.805

*adjusted for age, ethnolinguistic group, years of education, median family income, respondent status, cigarette index and IARC Group 1 known carcinogens (asbestos, diesel exhaust, formaldehyde, cadmium, chromium VI, nickel and silica).

Table 5 presents the results for each of the major histological types, for each study. Analyses were analogous to those reported for all lung cancers. For squamous cell carcinomas, we found an increased risk with substantial exposure, in study I with cancer controls (OR = 1.7, 95% CI = 1.1-2.6). We did not find elevated risks for small cell carcinomas in either study. For adenocarcinoma and large cell carcinoma, respectively, we found increased risk after substantial exposure to wood dust, only in study II (OR = 1.9, 95% CI = 1.0-3.7 and OR = 2.7, 95% CI = 1.2-6.0). However, smaller numbers of cases and controls in each sub-type analyses prevent us from drawing strong conclusions.

**Table 5:** **Odds ratio for lung cancer associated with occupational exposure to wood dust by histological types**

		Squamous cell				Small cell				Adenocarcinoma				Large cell and others			
	Controls	Cases	OR*	95% CI		Cases	OR*	95% CI		Cases	OR*	95% CI		Cases	OR*	95% CI	
**Study I**	*Population controls*																
No exposure	389	255	1.0	(ref)		116	1.0	(ref)		131	1.0	(ref)		128	1.0	(ref)	
Any level of exposure	144	104	0.8	0.5	1.2	43	0.7	0.4	1.2	36	0.5	0.3	0.8	44	0.7	0.4	1.1
Non-subst. level	74	49	0.8	0.5	1.2	19	0.6	0.3	1.2	21	0.7	0.4	1.2	24	0.7	0.4	1.3
Substantial level	70	55	0.8	0.5	1.4	24	0.8	0.4	1.5	15	0.3	0.2	0.7	20	0.6	0.3	1.1
**Study I**	*Cancer Controls*																
No exposure	1072	255	1.0	(ref)		116	1.0	(ref)		131	1.0	(ref)		128	1.0	(ref)	
Any level of exposure	272	104	1.3	0.9	1.8	43	1.2	0.7	1.8	36	1.0	0.6	1.5	44	1.1	0.7	1.7
Non-subst. level	161	49	1.0	0.7	1.5	19	0.9	0.5	1.5	21	1.0	0.6	1.7	24	1.0	0.6	1.7
Substantial level	116	55	1.7	1.1	2.6	24	1.6	0.9	2.8	15	0.9	0.5	1.7	20	1.2	0.7	2.1
**Study II**	*Population Controls*																
No exposure	640	166	1.0	(ref)		87	1.0	(ref)		171	1.0	(ref)		77	1.0	(ref)	
Any level of exposure	254	95	1.2	0.8	1.7	38	0.8	0.5	1.3	70	1.1	0.8	1.7	32	1.1	0.7	1.9
Non-subst. level	201	72	1.1	0.8	1.7	26	0.6	0.3	1.1	50	1.0	0.6	1.5	19	0.8	0.5	1.5
Substantial level	53	23	1.3	0.7	2.5	12	1.8	0.8	4.3	20	1.9	1.0	3.7	13	2.7	1.2	6.0

*adjusted for age, ethnolinguistic group, years of education, median family income, respondent status, cigarette index and IARC Group 1 known carcinogens (asbestos, diesel exhaust, formaldehyde, cadmium, chromium VI, nickel and silica).

To assess whether the inclusion of proxy responses influenced results, we conducted a sensitivity analysis restricted to self-respondents. The results are shown in a supplementary Table that mimics Table 3 [see Additional file 2: Table S2]. The ORs among self-respondents were similar to those found among all subjects. The OR for substantial exposure in study II, among self-respondents, was 1.6 (95% CI = 1.0-2.6), while among all subjects it was 1.7 (95% CI = 1.1-2.7).

## Discussion

Millions of people worldwide are exposed occupationally and non-occupationally to inhalable wood dust. Whereas there is sufficient evidence in humans for the carcinogenicity of wood dust on nasal cavity, paranasal sinuses and nasopharynx, evidence on the association with lung cancer remains inconclusive [5, 9, 33]. From an attributable risk or a compensation point of view, it is more important to establish whether or not wood dust causes lung cancer. Among studies that reported an increase in risk of lung cancer and where tobacco smoking was adjusted for, the exposure circumstances included pulp-paper mill workers [10, 16], woodworking [14, 34], furniture or cabinet-maker [35] or varied sources of exposure [6, 15, 17, 33, 36]. Many previous studies focused on a particular industry or locale where it could be expected that exposure levels were quite high. We believe that our population-based study sample included a broader range of exposure circumstances than most previous studies, and that there were proportionately more subjects with lower exposure levels than in some previously studied cohorts.

When all wood-exposed workers were combined, we found no clear association between occupational exposure to wood dust and lung cancer in the analyses of our three subsets. However, among those exposed to wood dust at a substantial cumulative level, we found a statistically significant risk in study II, and in study I when using cancer controls. Overall relative risks seemed slightly higher among never-low smokers than among medium-heavy smokers, but this effect modification was not statistically significant. To the extent that such effect modification has been previously explored, some investigators found the higher risk of wood dust related lung cancer among smokers than among non-smokers [6, 17, 33].

We included in the final models all variables that were considered as known or *a priori* confounders. A sensitivity analysis evaluating the impact of inclusion or exclusion of SES and other occupational carcinogens indicated that in this study, inclusion of SES, after including smoking and other a priori confounders, did not affect the results for wood dust. The same was mainly true in regard to inclusion of other occupational carcinogens, though in Study II there was a small but distinct reduction in OR when other occupational carcinogens were included. In themselves, these sensitivity analyses do not answer the question as to whether SES is a confounder or a collider.

The elevated risk of lung cancer among workers with substantial exposure was also found for squamous cell cancers, large cell carcinomas and adenocarcinomas, but not for small cell carcinomas. Some other investigators have also reported stronger associations between wood dust and squamous cell [13, 17], adenocarcinoma [33] and non-small cell carcinomas [15] than small cell carcinomas.

It is not clear why results in study I differed when using population controls or cancer controls. ORs were lower using population controls, reflecting a higher prevalence of exposed subjects among population controls (27% overall and 13% at the substantial level) than among cancer controls (21% overall and 9% at the substantial level). We postulate that the lower participation rate among population controls (72%) than among cancer controls (80%) has produced a biased set of population controls, but we cannot be certain of this conjecture.

The era of exposure spanned several decades, mainly from 1940s to 1970s for study I, and from 1960s to 1990s in study II. Because of the substantial overlap in eras of exposure between the two studies, it is difficult to use the trend in results between the two studies to draw inference about the changing risks over time. As seen in Table 2, there were some shifts over time in the distribution of occupations exposed to wood dust. Study II had more construction workers than Study I, whereas Study I had more timber cutters than Study II.

Strengths of this work include the large number of subjects, the availability of histological type of lung cancer, the collection of detailed lifetime job histories, the labor-intensive expert assessment of exposure, and the collection of extensive information on smoking and other covariates. In addition to being able to carefully control for the possible confounding effect of smoking [28], we were also able to control for major occupational carcinogenic co-exposures. Occupational exposure was attributed retrospectively to subjects on the basis of their lifetime job history reported at the interview and their assessment by a team of experts. We have previously shown that subjects’ reports of occupational history were valid [37] and that our team of chemists and industrial hygienists attributed exposure with reasonable reliability [38] and validity [39]. Nevertheless, our study is subject to exposure misclassification; this misclassification is likely to be non-differential between cases and controls since assessment of exposure was done blindly with respect to disease status. Other limitations are the inability to assess whether exposures were to hardwoods or softwoods and the lack of quantitative data on exposure levels.

## Conclusion

In conclusion, we did not find a clear association between occupational exposure to wood dust and lung cancer, when all wood-exposed workers were grouped together. When restricting to those exposed to wood dust at a substantial level, we found an increased risk in two of three subsets of the data. These results provide evidence in favor of the hypothesis of an association between substantial exposure to wood dust and lung cancer. Given the high prevalence of exposure to wood dust, these results are important.

## Electronic supplementary material

Additional file 1: Table S1: Odds ratio for lung cancer associated with occupational exposure to wood dust in two case–control studies, with and without adjustment for markers of SES and coexposure to Group 1 carcinogens. (DOC 66 KB)

Additional file 2: Table S2: Odds ratio for lung cancer associated with occupational exposure to wood dust in two case–control studies, restricted to self-respondents. (DOC 48 KB)
