# Supplementary material for: Occupational exposure to wood dust and risk of lung cancer in two population-based case–control studies in Montreal, Canada
Source: Environ Health. 2015 Jan 7;14:1. doi: 10.1186/1476-069X-14-1 (PMC4417249; doi:10.1186/1476-069X-14-1)
Supplement: Supplementary file 1 — Additional file 1: Table S1: Odds ratio for lung cancer associated with occupational exposure to wood dust in two case–control studies, with and without adjustment for markers of SES and coexposure to Group 1 carcinogens. (DOC 66 KB) [file 12940_2014_839_MOESM1_ESM.doc]

Additional file 1: Table S1: Odds ratio for lung cancer associated with occupational exposure to wood dust in two case-control studies, with and without adjustment for markers of SES and for co-exposure to IARC Group 1 occupational carcinogens

| **Study I (1979-86)** | **Population Controls** | **Cases** | **OR1*** | **95% CI** | | **OR2**** | **95% CI** | | **OR3***** | **95% CI** | | **OR4****** | **95% CI** | |
| --- | --- | --- | --- | --- | --- | --- | --- | --- | --- | --- | --- | --- | --- | --- |
| No exposure | 389 | 630 | 1.0 | (ref) | | 1.0 | (ref) | | 1.0 | (ref) | | 1.0 | (ref) | |
| Any level of exposure | 144 | 227 | 0.9 | 0.7 | 1.2 | 0.8 | 0.6 | 1.0 | 0.8 | 0.6 | 1.1 | 0.7 | 0.5 | 1.0 |
| Non-substantial level | 74 | 113 | 0.9 | 0.6 | 1.3 | 0.8 | 0.5 | 1.1 | 0.8 | 0.6 | 1.2 | 0.7 | 0.5 | 1.0 |
| Substantial level | 70 | 114 | 0.9 | 0.6 | 1.3 | 0.7 | 0.5 | 1.1 | 0.8 | 0.5 | 1.2 | 0.7 | 0.5 | 1.0 |
| **Study I (1979-86)** | **Cancer Controls** | **Cases** | **OR1** | **95% CI** | | **OR2** | **95% CI** | | **OR3** | **95% CI** | | **OR4** | **95% CI** | |
| No exposure | 1072 | 630 | 1.0 | (ref) | | 1.0 | (ref) | | 1.0 | (ref) | | 1.0 | (ref) | |
| Any level of exposure | 277 | 227 | 1.3 | 1.0 | 1.6 | 1.2 | 1.0 | 1.5 | 1.2 | 0.9 | 1.5 | 1.1 | 0.9 | 1.5 |
| Non-substantial level | 161 | 113 | 1.1 | 0.8 | 1.4 | 1.0 | 0.8 | 1.4 | 1.0 | 0.8 | 1.3 | 1.0 | 0.7 | 1.3 |
| Substantial level | 116 | 114 | 1.6 | 1.2 | 2.1 | 1.5 | 1.1 | 2.0 | 1.4 | 1.0 | 2.0 | 1.4 | 1.0 | 2.0 |
| **Study II (1996-2001)** | **Population Controls** | **Cases** | **OR1** | **95% CI** | | **OR2** | **95% CI** | | **OR3** | **95% CI** | | **OR4** | **95% CI** | |
| No exposure | 640 | 501 | 1.0 | (ref) | | 1.0 | (ref) | | 1.0 | (ref) | | 1.0 | (ref) | |
| Any level of exposure | 254 | 235 | 1.2 | 1.0 | 1.6 | 1.2 | 1.0 | 1.6 | 1.1 | 0.8 | 1.4 | 1.1 | 0.9 | 1.5 |
| Non-substantial level | 201 | 167 | 1.1 | 0.8 | 1.4 | 1.1 | 0.8 | 1.4 | 1.0 | 0.7 | 1.3 | 1.0 | 0.7 | 1.3 |
| Substantial level | 53 | 68 | 1.8 | 1.2 | 2.9 | 1.9 | 1.2 | 3.0 | 1.6 | 1.0 | 2.6 | 1.7 | 1.1 | 2.7 |

* Adjusted for age, ethno-linguistic group, respondent status and cigarette index. Not adjusted for SES (years of education and median family income) or for IARC Group 1 carcinogens.

** Adjusted for age, ethno-linguistic group, respondent status and cigarette index. Adjusted for SES, not adjusted for IARC Group 1 carcinogens.

*** Adjusted for age, ethno-linguistic group, respondent status and cigarette index. Not adjusted for SES, adjusted for IARC Group 1 carcinogens.

**** Adjusted for age, ethno-linguistic group, respondent status and cigarette index. Adjusted for SES and for IARC Group 1 carcinogens.
