# Supplementary material for: Occupational exposure to wood dust and risk of lung cancer in two population-based case–control studies in Montreal, Canada
Source: Environ Health. 2015 Jan 7;14:1. doi: 10.1186/1476-069X-14-1 (PMC4417249; doi:10.1186/1476-069X-14-1)
Supplement: Supplementary file 2 — Additional file 2: Table S2: Odds ratio for lung cancer associated with occupational exposure to wood dust in two case–control studies, restricted to self-respondents. (DOC 48 KB) [file 12940_2014_839_MOESM2_ESM.doc]

Additional file 2: Table S2: Odds ratio for lung cancer associated with occupational exposure to wood dust in two case-control studies, restricted to self-respondents

|  | **Study I (1979-86)** | | | | | | | | | | **Study II (1996-2001)** | | | | |
| --- | --- | --- | --- | --- | --- | --- | --- | --- | --- | --- | --- | --- | --- | --- | --- |
|  | **Population Controls** | **Cases** | **OR** | **95% CI** | | **Cancer Controls** | **Cases** | **OR** | **95% CI** | | **Population Controls** | **Cases** | **OR** | **95% CI** | |
| No exposure | 344 | 429 |  |  |  | 847 | 429 |  |  |  | 576 | 276 |  |  |  |
| Any level of exposure | 122 | 176 | 0.7 | 0.5 | 1.0 | 243 | 176 | 1.1 | 0.8 | 1.4 | 231 | 167 | 1.1 | 0.8 | 1.5 |
| Any level ≤ 20 years | 77 | 111 | 0.7 | 0.5 | 1.1 | 163 | 111 | 1.0 | 0.7 | 1.4 | 150 | 108 | 1.1 | 0.8 | 1.5 |
| Any level > 20 years | 45 | 65 | 0.8 | 0.5 | 1.3 | 80 | 65 | 1.3 | 0.8 | 1.9 | 81 | 59 | 1.2 | 0.8 | 1.9 |
| Non-substantial level | 64 | 90 | 0.8 | 0.5 | 1.2 | 143 | 90 | 0.9 | 0.7 | 1.3 | 181 | 117 | 1.0 | 0.7 | 1.4 |
| Substantial level | 58 | 86 | 0.7 | 0.5 | 1.1 | 100 | 86 | 1.4 | 0.9 | 2.0 | 50 | 50 | 1.6 | 1.0 | 2.6 |

*adjusted for age, ethno-linguistic group, years of education, median family income, cigarette index and IARC Group 1 carcinogens (asbestos, diesel exhaust, formaldehyde, cadmium, chromium VI, nickel and silica)
